# Facioscapulohumeral Dystrophy: Incomplete Suppression of a Retrotransposed Gene

**DOI:** 10.1371/journal.pgen.1001181

**Published:** 2010-10-28

**Authors:** Lauren Snider, Linda N. Geng, Richard J. L. F. Lemmers, Michael Kyba, Carol B. Ware, Angelique M. Nelson, Rabi Tawil, Galina N. Filippova, Silvère M. van der Maarel, Stephen J. Tapscott, Daniel G. Miller

**Affiliations:** 1 Division of Human Biology, Fred Hutchinson Cancer Research Center, Seattle, Washington, United States of America; 2 Department of Human Genetics, Leiden University Medical Center, Leiden, The Netherlands; 3 Lillehei Heart Institute and Department of Pediatrics, University of Minnesota, Minneapolis, Minnesota, United States of America; 4 Department of Comparative Medicine, University of Washington, Seattle, Washington, United States of America; 5 Department of Neurology, University of Rochester, Rochester, New York, United States of America; 6 Department of Neurology, University of Washington, Seattle, Washington, United States of America; 7 Department of Pediatrics, University of Washington, Seattle, Washington, United States of America; The Hospital for Sick Children and University of Toronto, Canada

## Abstract

Each unit of the D4Z4 macrosatellite repeat contains a retrotransposed gene encoding the DUX4 double-homeobox transcription factor. Facioscapulohumeral dystrophy (FSHD) is caused by deletion of a subset of the D4Z4 units in the subtelomeric region of chromosome 4. Although it has been reported that the deletion of D4Z4 units induces the pathological expression of DUX4 mRNA, the association of DUX4 mRNA expression with FSHD has not been rigorously investigated, nor has any human tissue been identified that normally expresses DUX4 mRNA or protein. We show that FSHD muscle expresses a different splice form of DUX4 mRNA compared to control muscle. Control muscle produces low amounts of a splice form of DUX4 encoding only the amino-terminal portion of DUX4. FSHD muscle produces low amounts of a DUX4 mRNA that encodes the full-length DUX4 protein. The low abundance of full-length DUX4 mRNA in FSHD muscle cells represents a small subset of nuclei producing a relatively high abundance of DUX4 mRNA and protein. In contrast to control skeletal muscle and most other somatic tissues, full-length DUX4 transcript and protein is expressed at relatively abundant levels in human testis, most likely in the germ-line cells. Induced pluripotent (iPS) cells also express full-length DUX4 and differentiation of control iPS cells to embryoid bodies suppresses expression of full-length DUX4, whereas expression of full-length DUX4 persists in differentiated FSHD iPS cells. Together, these findings indicate that full-length DUX4 is normally expressed at specific developmental stages and is suppressed in most somatic tissues. The contraction of the D4Z4 repeat in FSHD results in a less efficient suppression of the full-length DUX4 mRNA in skeletal muscle cells. Therefore, FSHD represents the first human disease to be associated with the incomplete developmental silencing of a retrogene array normally expressed early in development.

## Author Summary

Facioscapulohumeral muscular dystrophy is caused by the deletion of a subset of D4Z4 macrosatellite repeats on chromosome 4. Each repeat contains a retrogene encoding the double-homeobox factor DUX4. We show that this retrogene is normally expressed in human testis, most likely the germ-line cells, and pluripotent stem cells. DUX4 expression is epigenetically suppressed in differentiated tissues and the residual DUX4 transcripts are spliced to remove the carboxyterminal domain that has been associated with cell toxicity. In FSHD individuals, the expression of the full-length DUX4 transcript is not completely suppressed in skeletal muscle, and possibly other differentiated tissues, and results in a small percentage of cells expressing relatively abundant amounts of the full-length DUX4 mRNA and protein. We therefore propose that FSHD is caused by the inefficient developmental suppression of the DUX4 retrogene and that the residual expression of the full-length DUX4 in skeletal muscle is sufficient to cause the disease. Therefore, FSHD represents the first human disease to be associated with the incomplete developmental silencing of a retrogene array that is normally expressed early in development.

## Introduction

Facioscapulohumeral dystrophy (FSHD) is an autosomal dominant muscular dystrophy caused by the deletion of a subset of D4Z4 macrosatellite repeat units in the subtelomeric region of 4q on the 4A161 haplotype (FSHD1; OMIM 158900) [1]. The unaffected population has 11–100 D4Z4 repeat units, whereas FSHD1 is associated with 1–10 units [2]. The retention of at least a portion of the D4Z4 macrosatellite in FSHD1 and the demonstration that the smaller repeat arrays have diminished markings of heterochromatin [3] support the hypothesis that repeat contraction results in diminished heterochromatin-mediated repression of a D4Z4 transcript, or a transcript from the adjacent subtelomeric region. The hypothesis that derepression of a regional transcript causes FSHD is further supported by individuals with the same clinical phenotype and decreased D4Z4 heterochromatin markings but without a contraction of the D4Z4 macrosatellite in the pathogenic range (FSHD2) [4], [5].

The D4Z4 repeat unit contains a conserved open reading frame for the DUX4 retrogene, which Clapp et al suggest originated from the retrotransposition of the DUXC mRNA [6], a gene present in many mammals but lost in the primate lineage. Dixit et al [7] demonstrated that DUX4 transcripts were present in cultured FSHD muscle cells and mapped a polyadenylation site to the region telomeric to the last repeat, a region referred to as pLAM. Lemmers et al [8] recently demonstrated that the region necessary for a contracted D4Z4 array to be pathogenic maps to this polyadenylation site, which is intact on the permissive 4A chromosome but not on the non-permissive chromosomes 4B or 10, indicating that stabilization of the DUX4 mRNA is necessary to develop FSHD on a contracted allele. Our prior study [9] demonstrated bidirectional transcription of the D4Z4 region associated with the generation of small RNAs, and we suggested that these D4Z4-associated small RNAs might contribute to the epigenetic silencing of D4Z4. We also identified alternatively spliced transcripts from the DUX4 retrogene that terminate at the previously described [7] polyadenylation site in the pLAM region. However, we identified DUX4 mRNA transcripts in both FSHD and wild-type muscle cells, as well as similar amounts of D4Z4-generated small RNAs.

Together these studies implicate a stabilized DUX4 mRNA transcript from the contracted D4Z4 array as the cause of FSHD. However, several important questions remain to be addressed:

1. Our prior study identified two alternative splice forms of the DUX4 mRNA, which in this report we call DUX4-fl and DUX4-s, and showed that both control and FSHD muscle with a 4A chromosome contained polyadenylated DUX4 mRNA. Therefore, it is important to determine whether the overall abundance of the DUX4 mRNA or the relative abundance of the alternative splice forms is associated with FSHD.
2. All studies reporting DUX4 mRNA associated with FSHD have used high cycle PCR to detect mRNA that are present at extremely low abundance. It remains to be determined whether the amount of DUX4 mRNA detected in FSHD cells makes sufficient DUX4 protein to have a biological consequence.
3. DUX4 has been referred to as a pseudogene and the D4Z4 region has been referred to as “junk” DNA. The conclusion that DUX4 is not a functional gene is supported only by the absence of evidence that the DUX4 mRNA and protein is normally expressed in any human tissue. Yet, the open reading frame (ORF) of DUX4 is conserved, raising the possibility that it might have an as yet undetected role in human biology.

In this study, we address each of these important questions. Together, our data substantiate a developmental model for FSHD: full-length DUX4 mRNA is normally expressed early in development and is suppressed during cellular differentiation, whereas FSHD is associated with the failure to maintain complete suppression of full-length DUX4 expression in differentiated skeletal muscle cells. Occasional escape from repression results in the expression of relatively large amounts of DUX4 protein in a small number of skeletal muscle nuclei.

## Results

### Alternative DUX4 mRNA splicing distinguishes control and FSHD muscle

**Figure 1:**
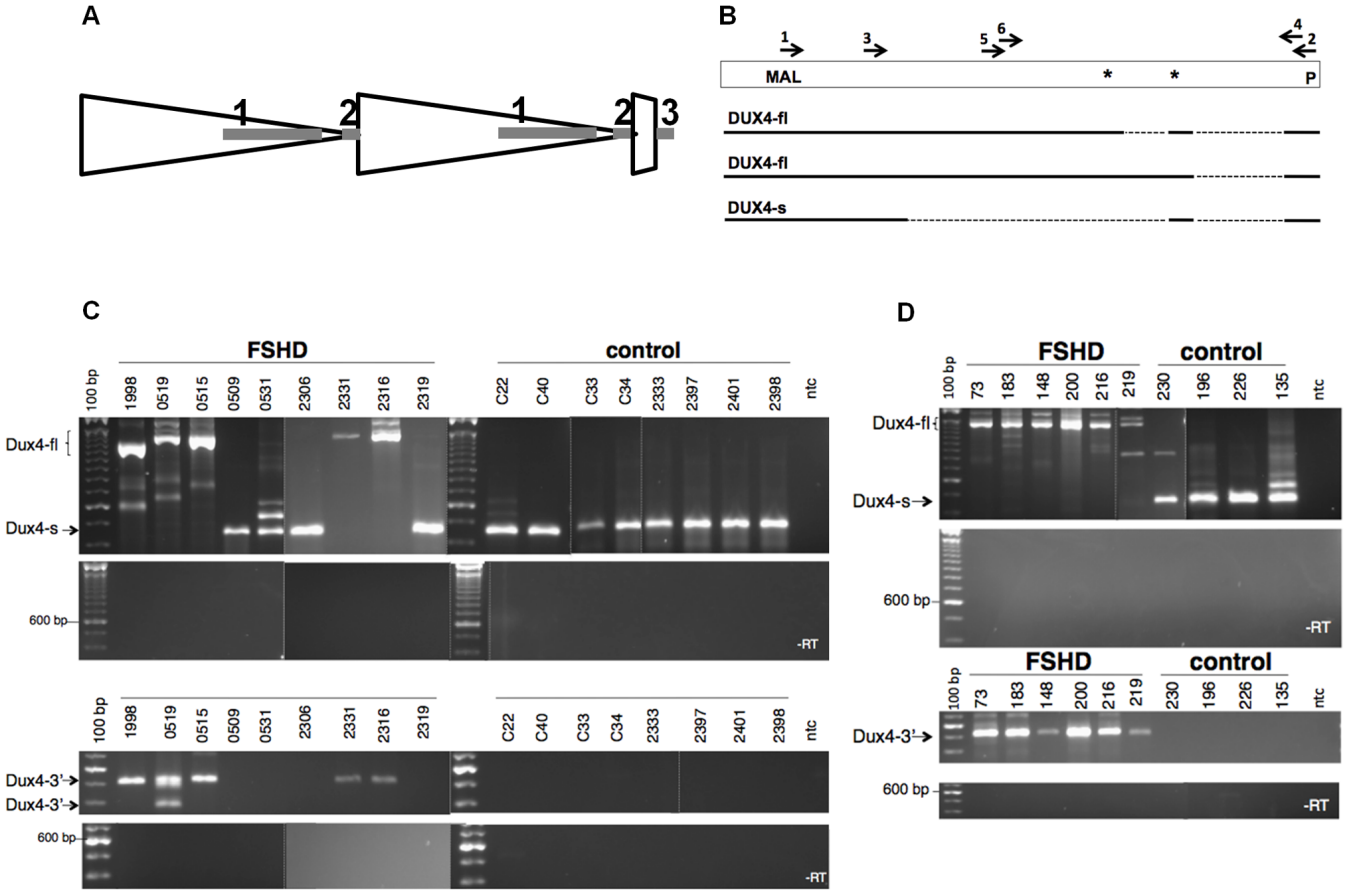
Expression of DUX4-fl and DUX4-s and D4Z4 in control and FSHD cells. (A) Diagram of D4Z4 repeat array with two most telomeric full units (large triangles), the last partial repeat, and the adjacent pLAM sequence that contains exon 3. Exons shown as shaded rectangles, with exon 1 and 2 in the D4Z4 units and exon 3 in the pLAM region. (B) The open rectangle represents the region of D4Z4 and pLAM containing the DUX4 retrogene, and the solid and dashed lines represent the regions of exons and introns, respectively, in the short splice form (DUX4-s) and the transcript with the full-length DUX4 ORF (DUX4-fl), which has two isoforms with alternative splicing in the 3-prime untranslated region. First round PCR for DUX4-fl and DUX4-s was performed with primer sets 1 and 2 and second round PCR with nested primers 3 and 4. Nesting was used to ensure specificity and because of the very low abundance of DUX4 transcripts, both DUX4-fl and DUX4-s. MAL, represents location of initial amino-acid codons; *, Stop codons; P, polyadentylation site. (C) Composite image of representative PCR products from FSHD and control muscle biopsies for DUX4-fl, DUX4-s, and DUX4-fl3′. DUX4-fl and DUX4-s are indicated. Variation in size reflects alternative intron usage and the faint intermediate bands represent background non-DUX4 PCR amplicons frequently associated with repetitive sequence. (D) Representative PCR products of cultured FSHD and control muscle cells under differentiation conditions. ntc, no template control.

A recent study [8] demonstrated that the sequence polymorphisms of the 4A161 haplotype necessary for FSHD include the region of the poly-adenylation signal for the DUX4 mRNA and showed that this correlated with the detection of DUX4 mRNA in three FSHD muscle cultures compared to controls. Our previous study of RNA transcripts from D4Z4 repeat units identified a full-length mRNA transcript that contains the entire DUX4 open reading frame and has one or two introns spliced in the 3-prime UTR (GenBank HQ266760 and HQ266761), and a second mRNA transcript utilizing a cryptic splice donor in the DUX4 ORF that maintains the amino-terminal double-homeobox domains and removes the carboxyterminal end of DUX4 (GenBank HQ266762) (Figure 1A and 1B). We will refer to these two transcripts as DUX4-fl (full length) and DUX4-s (shorter ORF), respectively (see [9] for splice junction sequences). The PCR approach in the Lemmers et al study [8] would not have detected the DUX4-s mRNA.

We used oligo-dT primed cDNA and a PCR strategy that would detect both DUX4-fl and DUX4-s (see Figure 1B) to determine the presence of polyadenylated DUX4 mRNAs in quadriceps muscle needle biopsies from ten FSHD and fifteen control individuals (Table 1 and Figure 1C). In general we used two cycles of PCR with nested primers to increase specificity and to detect low abundance transcripts. DUX4-fl was detected in five of the ten FSHD samples, based on primers amplifying DUX4-fl and primers amplifying the 3-prime region of DUX4-fl (DUX4-fl3′) that is contained in DUX4-fl but not in DUX4-s (see Figure 1B). The sequenced products matched the FSHD-permissive 4A161 haplotype polymorphisms and the variation in size of the PCR product reflected alternative splicing of only the second intron in the UTR or both the first and second UTR introns (see Figure 1B). In contrast, none of the fifteen control samples expressed mRNA that amplified with primers to DUX4-fl or DUX4-fl3′, including seven biopsies from individuals with at least one 4A161 chromosome. Instead, DUX4-s was detected in all control samples with 4A161 and in some of the FSHD samples. We did not detect DUX4 transcripts using these primers in six control biopsies that do not contain the 4A chromosome. These data indicate that the 4A D4Z4 region is actively transcribed and produces alternatively spliced and polyadenylated DUX4 mRNA in both FSHD and unaffected individuals. However, the full-length DUX4 mRNA was only detected in the FSHD muscle biopsies, whereas DUX4-s was detected in muscle from controls and some FSHD individuals.

**Table 1:** DUX4 mRNA expression in FSHD and control biopsies.

Biopsy Code	Status	Haplotypes^A^	DUX4-fl	DUX4-s	3-prime
F 1998	FSHD	A161(10)/n.d.	**XS**	O	**XS**
F 0519	FSHD	A161(8)/A161	**XS**	O	**XS**
F-0515	FSHD	A161(5)/A161	**XS**	O	**XS**
F-0509	FSHD	A161(5)/A166H	O	**XS**	O
F-0531	FSHD	A161(7)/A161	O	**XS**	O
F-2306	FSHD	A161(4)/B163	O	X	O
F-2331	FSHD	A161(3)/B163	X	O	**XS**
F-2316	FSHD	A161(6)/A168	X	O	**XS**
F-2319	FSHD	A161(5)/B168	O	X	O
F-2315	FSHD	A161(5)/B168	O	O	O
C22	contr	A161/B163	O	X	O
C34	contr	A161/B163	O	**XS**	O
C40	contr	A161/B168	O	X	O
2333	contr	A161/A161	O	X	O
2397	contr	A161/B168	O	X	O
2401	contr	A161/B168	O	X	O
2398	contr	A161/B163	O	X	O
C33	contr	A166/B170	O	X	O
C39	contr	A166/B168	O	O	O
C10	contr	B168/B168	O	O	O
C11	contr	B168/B163	O	O	O
C31	contr	B163/B163	O	O	O
2318*	contr	B169/B166	O	O	O
C20	contr	B168/B168	O	O	O
C38	contr	B162/B163	O	O	O

A Chrosome 4 haplotype. For FSHD the number in paratheses indicates the number of D4Z4 units on the contracted allele; n.d., indicates that the haplotype of the second allele was not determined.

X, product present; **XS**, product sequenced; O, product absent.

*has contracted 10qA allele with 9 repeats.

The expression of DUX4-fl mRNA in FSHD muscle biopsies could be a primary consequence of the D4Z4 contraction or a secondary response to the inflammation associated with muscle degeneration and/or regeneration. Therefore, we extended our analysis to myoblast cultures derived from four control and six FSHD individuals, including one individual with FSHD2. As seen in the muscle biopsies, the control muscle cells contained no detectable amounts of DUX4-fl mRNA, whereas muscle cells derived from both FSHD1 and FSHD2 samples expressed DUX4-fl transcripts as well as the DUX4fl-3′ (Table 2 and Figure 1D). All control and a subset of the FSHD samples expressed DUX4-s. These data are consistent with observations made in the muscle biopsies and indicate that both FSHD and control muscle cells actively transcribe DUX4. Unaffected cells produce DUX4-s from a splice donor site in the DUX4 ORF, whereas FSHD cells produce DUX4-fl with an alternative splice donor site after the translation termination codon of the DUX4 ORF.

**Table 2:** DUX4 mRNA expression in FSHD and control cell lines.

Cell Code	Status	Haplotypes	Cell type A	DUX4-fl	DUX4-s	3-prime
MB73	FSHD	A161(8)^B^/161^C^	MB	X	X	X
MB183	FSHD	A161(5)/B163	MB	X	O	X
MB148	FSHD	A161(3)/A161	MB	X	O	X
MB200	FSHD2	A161(14)/B168	MB	X	**XS**	X
MB216	FSHD	A161(6)/A168	MB	O	O	O
MB219	FSHD	A161(5)/B168	MB	O	O	O
MB230	contr	A161/163^A^	MB	O	X	O
MB196	contr	A161/163^A^	MB	O	X	O
MB226	contr	A161/A161	MB	O	X	O
MB135	contr	A161/B163	MB	O	**XS**	O
MB73	FSHD	A161/161	MT	X	X	**XS**
MB183	FSHD	A161/B163	MT	X	O	X
MB148	FSHD	A161/A161	MT	X	O	**XS**
MB200	FSHD2	A161/B168	MT	**XS**	O	**XS**
MB216	FSHD	A161/A168	MT	X	O	**XS**
MB219	FSHD	A161/B168	MT	X	O	X
MB230	contr	A161/163^A^	MT	O	X	O
MB196	contr	A161/163^A^	MT	O	X	O
MB226	contr	A161/A161	MT	O	X	O
MB135	contr	A161/B163	MT	O	X	O
M83-9	contr	A161/168^A^	fibro	O	**XS**	O
M83-9	contr		iPS	**XS**	O	X
M83-9	contr		EB	O	X	O
43-1	FSHD	A161(5)/A161	fibro	**XS**	O	O
43-1	FSHD		iPS	**XS**	O	X
43-1	FSHD		EB	X	O	**XS**
83-6	FSHD	A161(7)/B168	fibro	**XS**	**XS**	X
83-6	FSHD		iPS	X	O	X
83-6	FSHD		EB	X	O	**XS**

A MB, myoblasts; MT, myotubes; fibro, fibroblasts; iPS, induced pleuripotent stem cells, EB, embryoid bodies.

B Number of repeats on contracted allele, if known.

C Assignment of the second 4q allele variant is incomplete.

n.d., not tested; X, product present; **XS**, product sequenced; O, product absent.

### A small fraction of FSHD muscle cells produce a relatively large amount of DUX4

In both control and FSHD cells the DUX4 mRNA transcripts, either DUX4-fl or DUX4-s, were only detected after nested PCR amplifications, indicating very low abundance of DUX4 mRNA in the FSHD and control biopsies and cells. We used the 9A12 mouse monoclonal anti-DUX4 antibody [7] and also produced mouse and rabbit monoclonal antibodies to the amino-terminal and carboxyterminal portion of the DUX4 protein [10], but were unable to detect DUX4 protein in western analysis of FSHD muscle cultures, consistent with the very low amounts of DUX4 mRNA.

**Figure 2:**
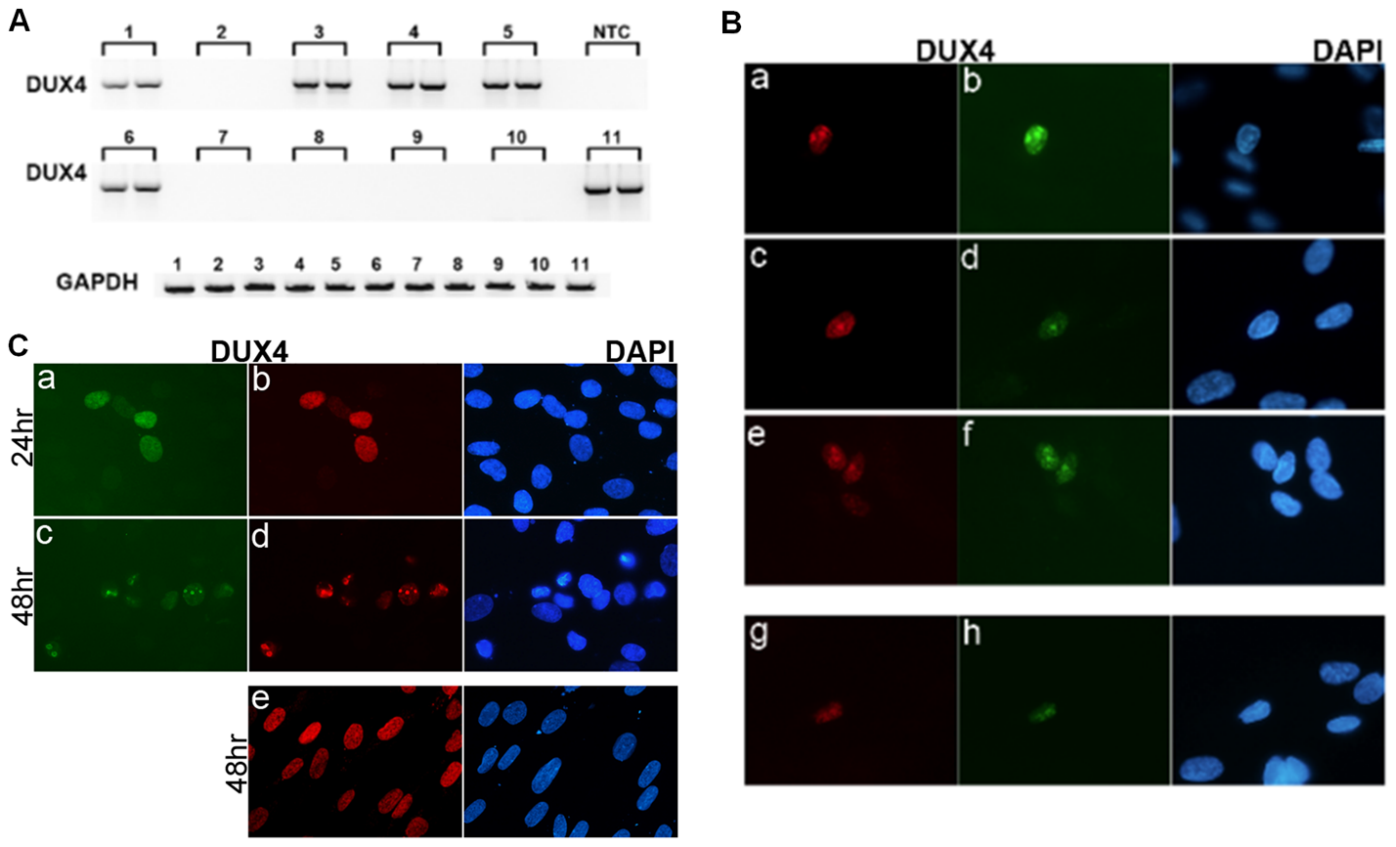
A small number of FSHD muscle cells express a relatively large amount of DUX4. (A) RT-PCR for full length DUX4 (DUX4-fl3′) was performed in duplicate on polyadenylated RNA isolated from ten pools of 600 cultured FSHD muscle cells (lanes 1–10) and a single pool of 10,000 cells (lane 11). The presence of DUX4 mRNA in one-half of the pools indicates that approximately one cell per 1000 is expressing DUX4 mRNA. NTC, no template control. (B) Cultured FSHD muscle cells were differentiated and immunostained with monoclonal antibodies to DUX4. Cells were co-stained with the E5-5 rabbit monoclonal antibody to the DUX4 C-terminal region (panels a, c, e) and the P2G4 mouse monoclonal antibody to the N-terminal region (panels b,d,f), or co-stained with the P4H2 mouse monoclonal antibody to the C-terminal region and the E14-3 rabbit monoclonal antibody to the N-terminal region. Approximately 1 cell per 1000 showed nuclear staining and the co-localization of both the n-terminal and c-terminal regions indicates that these cells are expressing the full-length DUX4 protein. No positive nuclei were apparent in the control muscle cultures. (C) Control human muscle cultures infected with a lenti-virus expressing full length DUX4 (a–d) or DUX4-s (e) co-stained with P4H2 (panels a, c) and E14-3 (panels b, d, e). In the cells expressing DUX4-fl, at 24 hrs there is a relatively homogenous nuclear distribution of DUX4 (a,b), whereas at 48 hrs as the cells are undergoing apoptosis DUX4 staining becomes more focal and punctate (c,d), similar to the punctate DUX4 staining in the FSHD muscle cells in panel B. Expression of DUX4-s does not induce apoptosis (data not shown) and the DUX4-s protein maintains a relatively homogenous nuclear distribution at 48 hrs (e).

Low transcript abundance could reflect a small number of transcripts in every cell or a large number of transcripts in a small subset of cells in the population. We assessed the presence of DUX4-fl mRNA in samplings of 100, 600, and 10,000 FSHD cultured muscle cells. DUX4-fl mRNA was present in five-out-of-ten pools of 600 cells (Figure 2A) and three-out-of-20 pools of 100 cells (data not shown), as well as in the single pool of 10,000 cells. This frequency of positive pools indicates that approximately one-out-of-1000 cells is expressing a relatively abundant amount of DUX4-fl mRNA at any given time. Immunostaining of cultured FSHD and control cultured muscle cells with four independent anti-DUX4 monoclonal antibodies showed that approximately one-out-of-1000 nuclei co-stained with an antibody to the amino-terminus and an antibody to the carboxy-terminus of DUX4 (Figure 2B), whereas no nuclei in the control cultures showed double-positive staining.

Both the mRNA analysis and the immunodetection indicate that approximately 0.1% of FSHD muscle nuclei express DUX4 mRNA and protein. This could represent transient bursts of expression or stochastic activation of expression that leads to cell death, or both. Forced expression of DUX4 has been shown to induce apoptosis in muscle cells [9], [11], [12]. When DUX4 is expressed in control human muscle cells by lenti-viral delivery, the DUX4 protein is distributed relatively homogeneously during the first 24 hrs and then aggregates in nuclear foci at 48 hrs when the cells are undergoing apoptosis (Figure 2C, panels c and d). These DUX4 nuclear foci associated with apoptosis are present in the nuclei of FSHD muscle cultures (compare panel d in Figure 2C with panels a–f in Figure 2B). Expression of DUX4-s in control human muscle cells does not induce apoptosis and does not accumulate in nuclear foci at 48 hrs (Figure 2, panel e). Therefore, the data indicates that FSHD muscle cells that express endogenous full-length DUX4 also exhibit the nuclear foci that are characteristic of DUX4-induced apoptosis.

### DUX4 mRNA and protein are expressed in human testis

Although there is no known function of DUX4 in human biology, the open reading frame has been conserved [6]. DUX4 is a retrogene thought to be derived from DUXC [6], or a DUXC-related gene, but also similar to the DUXA family mouse Duxbl gene [13]. Therefore, if DUX4 has a biological function it is likely to be similar to DUXC or Duxbl. Duxbl is expressed in mouse germ-line cells and we reasoned that because retrotranspositions entering the primate lineage must have occurred in the germ-line, then the parental gene to DUX4, either Duxbl or DUXC, must be expressed in the germ-line. Indeed, we detect the canine DUXC mRNA in canine testis but not in canine skeletal muscle (data not shown). Therefore, if DUX4 has a biological function similar to DUXC or Duxbl, we would anticipate DUX4 expression in the human germ-line.

**Figure 3:**
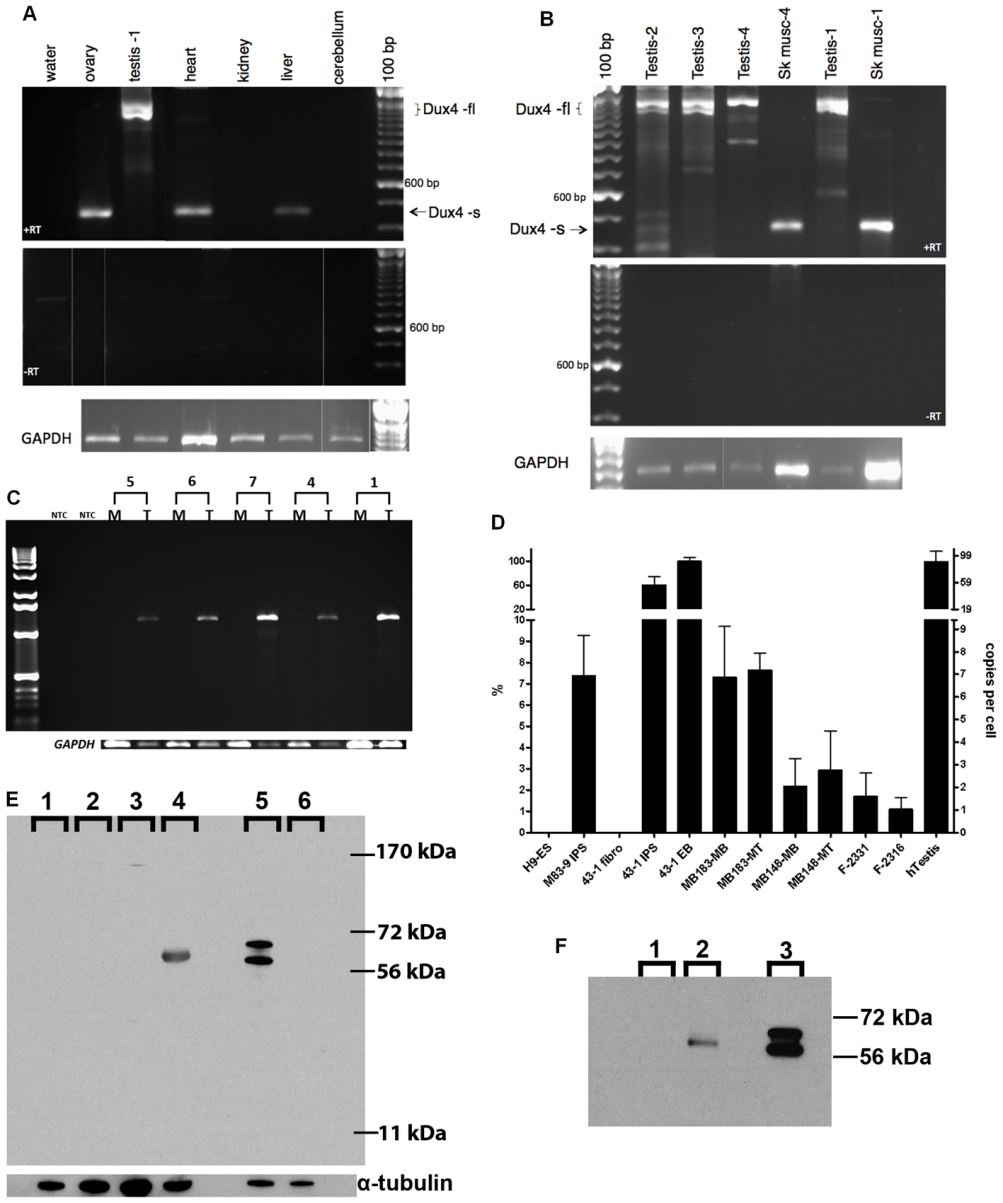
Expression of DUX4-fl in human tissues. (A) RT-PCR of RNA from human tissues showing DUX4-fl in the testis sample (Testis-1) and DUX4-s in ovary heart and liver. Note that each sample is from an unknown individual and their genotype is not known. (B) RT-PCR of three additional testis samples and matched skeletal muscle RNA from the Testis-1 and Testis-4 donors. (C) RT-PCR of the full-length DUX4 ORF (nested with 36 total cycles) in muscle (M) and testes (T) RNA from the same individuals, showing expression in testes and not in muscle. Numbers indicate Testis-1, 4, 7, 6, 5. (D) Quantitative RT-PCR of DUX4-fl3′ showing relative abundance in muscle cells, muscle biopsies, human testis and othr indicated cells. The sample with the highest expression was set at 100% and the number of copies per cell roughly estimated based on titration of input DNA. H9-ES, human embryonic cell line H9; M83-9 iPS, iPS line made from control fibroblasts; 43-1 series are the fibroblast, iPS, and EB cells from an FSHD fibroblast line; MB183 and MB148, FSHD muscle cultures under growth (MB) and differentiation (MT) conditions; F-2331 and F-2316, FSHD muscle biopsy samples; hTestis, human testis. (E) Western detection of DUX4 protein in whole cell extracts from tissues and cell lines using a rabbit monoclonal antibody (E14-3) raised to the aminoterminus of the human DUX4 protein. Lanes: 1-control muscle culture; 2-HCT116 cell line; 3-mouse testis; 4-human testis; 5-C2C12 cells transfected with human DUX4 expression vector; 6-C2C12 cells. Specificity of the antibody is indicated by selective detection of DUX4 protein in C2C12 cells transfected with a DUX4 expression vector, pCS2-DUX4 (note that this vector contains two ATG codons resulting in the standard DUX4 protein and an in-frame slightly larger protein accounting for the two bands on the western), compared to untransfected C2C12. Human testis has a single reactive band that migrates marginally slower than the transfected standard DUX4 species in C2C12 cells. This DUX4-reactive band is not present in mouse testis extract (note that mice do not have a highly conserved DUX4), HCT116 human colon cancer cells, nor unaffected myotubes (MB196-36hr). Similar results were obtained with an additional DUX4 antibody (E5-5) raised to the carboxyterminal region of DUX4 (data not shown)) and on protein extracts from two additional testis samples (data not shown). (F) Immunoprecipitation of indicated protein extracts with the E14-3 rabbit monoclonal to the N-terminal region of DUX4 followed by western with the P4H2 mouse monoclonal to the C-terminal region of DUX4 demonstrating that the protein recognized by the rabbit anti-DUX4 is also recognized by an independent mouse monoclonal to DUX4. Lanes: 1-HCT116 cell line lysate; 2- Testis protein lysate; 3-C2C12 cells transfected with DUX4 expression vector.

**Figure 4:**
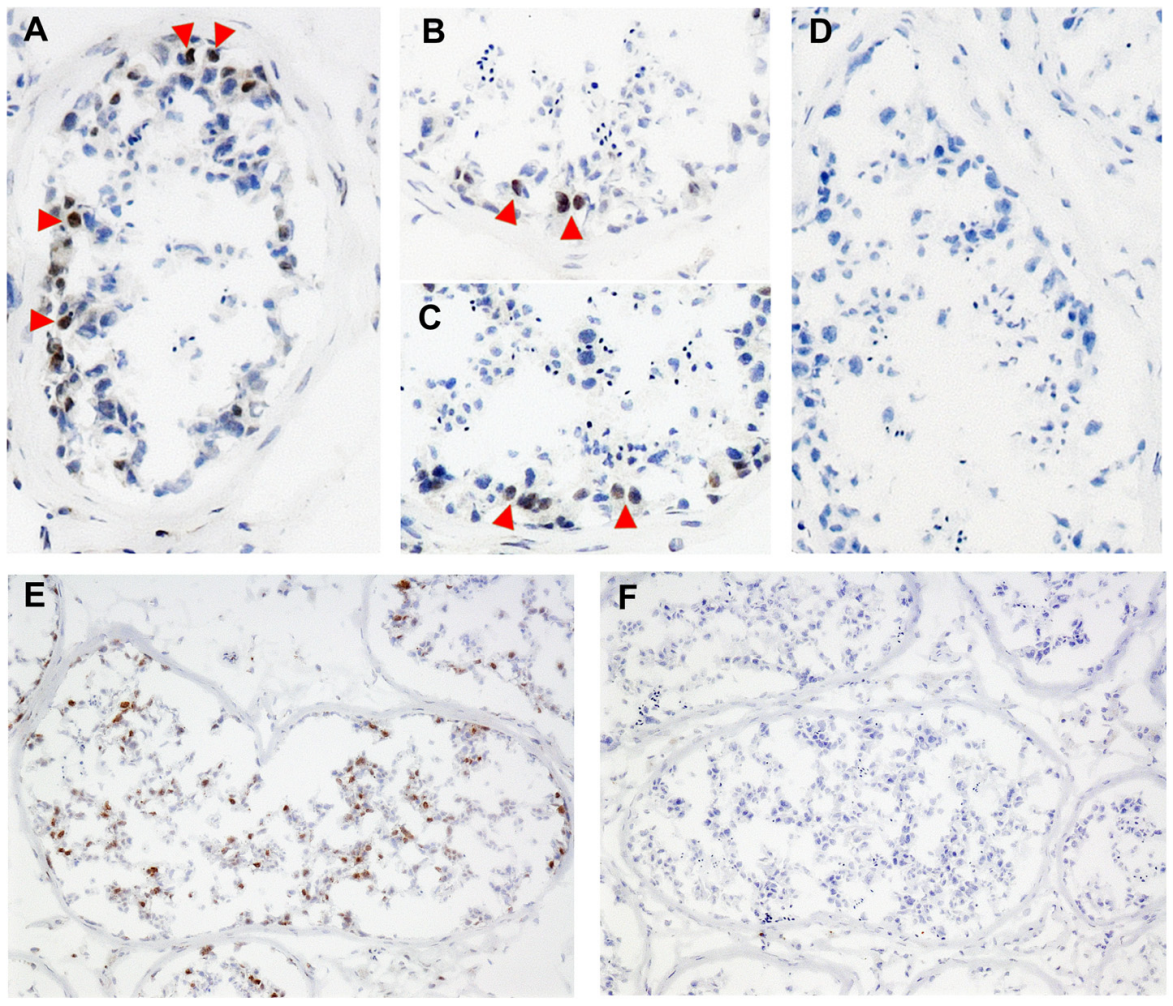
Expression of DUX4 in the testis. Frozen sections of human testis stained with anti-DUX4 monoclonal antibodies P2B1 and E5-5 together (A), or P2B1 (B), or E5-5 (C) individually, compared to an isotype control (D). Arrowheads indicate a few of the many nuclei that show a brown antibody-dependent precipitate superimposed on the blue hematoxylin stained nuclei. (E) Antigen retrieval reveals a larger number of more differentiated germ-line cells stained with the P2B1 antibody, compared to an isotype control (F). Because of the range of nuclear morphologies and relatively large numbers of positive cells, the DUX4 expressing cells in the germ line lineage at a range of differentiation stages.

We obtained RNA from different adult human tissues and identified DUX4-fl in testis (Figure 3A), whereas DUX4-s was present in a subset of differentiated tissues. DUX4-fl was detected in six additional testis samples, whereas only DUX4-s was detected in donor-matched skeletal muscle (Figure 3B and 3C). Quantitative PCR (qPCR) showed that human testis samples expressed almost 100-fold higher amounts of DUX4 mRNA compared to FSHD muscle biopsies, and almost 15-fold higher amounts compared to cultured FSHD muscle cells (Figure 3D). Western analysis using three different DUX4 antibodies identified a protein of the correct mobility in protein lysates from testes but not in other cells or tissues that do not express DUX4-fl mRNA, including control muscle cells (Figure 3E and data not shown). Furthermore, immunoprecipitation of testis proteins with rabbit anti-DUX4 antibodies followed by western with a mouse monoclonal antibody to DUX4 detected the same protein (Figure 3F). Western analysis of protein extracts from three additional human testis samples identified a similar band (data not shown). Immunostaining identified DUX4-expressing cells near the periphery of the seminiferous tubule that have the large round nucleus characteristic of spermatogonia or primary spermatocytes (Figure 4A–4C), and additional more differentiated appearing cells in the seminiferous tubules were also stained following antigen retrieval (Figure 4E). The large numbers and nuclear morphology of the cells staining with DUX4 in the seminiferous tubules, together with expression of DUX4 in the human germ-cell cell tumor lines SuSa and 833K [14] (data not shown), leads us to conclude that DUX4 is expressed in the germ-line lineage. Further studies will be necessary to determine more precisely the timing and cell stages of DUX4 expression in the in the testis and to ascertain whether it has a biological function.

### Chromosomes 4 and 10 produce DUX4 mRNA in human testes

The relatively high abundance of DUX4 mRNA and protein in human testes suggests a possible role for this protein in normal development. However, we have previously demonstrated that the alleles of chromosome 4 and 10 that are non-permissive for FSHD contain polymorphisms that inhibit polyadenylation of the DUX4 transcript, and, therefore, only the 4A allele would be predicted to make a DUX4 mRNA [8]. We do not have haplotype information on the testis donors and it is possible that some might lack the 4A haplotype entirely. To determine whether only the 4A haplotype produced stable DUX4 mRNA in human testes, we sequenced mRNAs from the seven testis samples in a region with informative polymorphisms regarding transcripts from 4A, 4B, and 10. All testis mRNA had transcripts from both chromosomes 4 and 10 in approximately equal amounts (Table 3) based on the informative polymorphisms (Table 4). Some samples had 4A and 4B haplotypes.

**Table 3:** Haplotype identification of DUX4 mRNA in human testis.

Sample Code	%10A A	% 4A	% 4B	Total Number B
T1	22	78	0	9
T2	60	40	0	10
T3: 8606	22	67	11	9
T4: H12817	56	0	44	9
T7: N30	56	22	22	9
T6: N21	20	80	0	10
T5: N11	22	78	0	9
Total	38	53	9	64

A Percentage of the sequences containing SNPs of each haplotype, see Table 4 for sequence polymorphisms.

B Number of sequenced cDNA.

**Table 4:** Diagnostic polymorphisms in exon 2.

4A161, 159, 168	GTCTAGGCCCGGTGAGAGACTCCACACCGCG
4A166	GTCTAGGCCCGGTGAGAGACTCCACACAGCG
10A166	GCCTAGGCCCGGTGAGAGACTCCACACAGCG
4B163	GTCCAGGCCCGGTGAGAGACTCCACACCGCG

**Figure 5:**
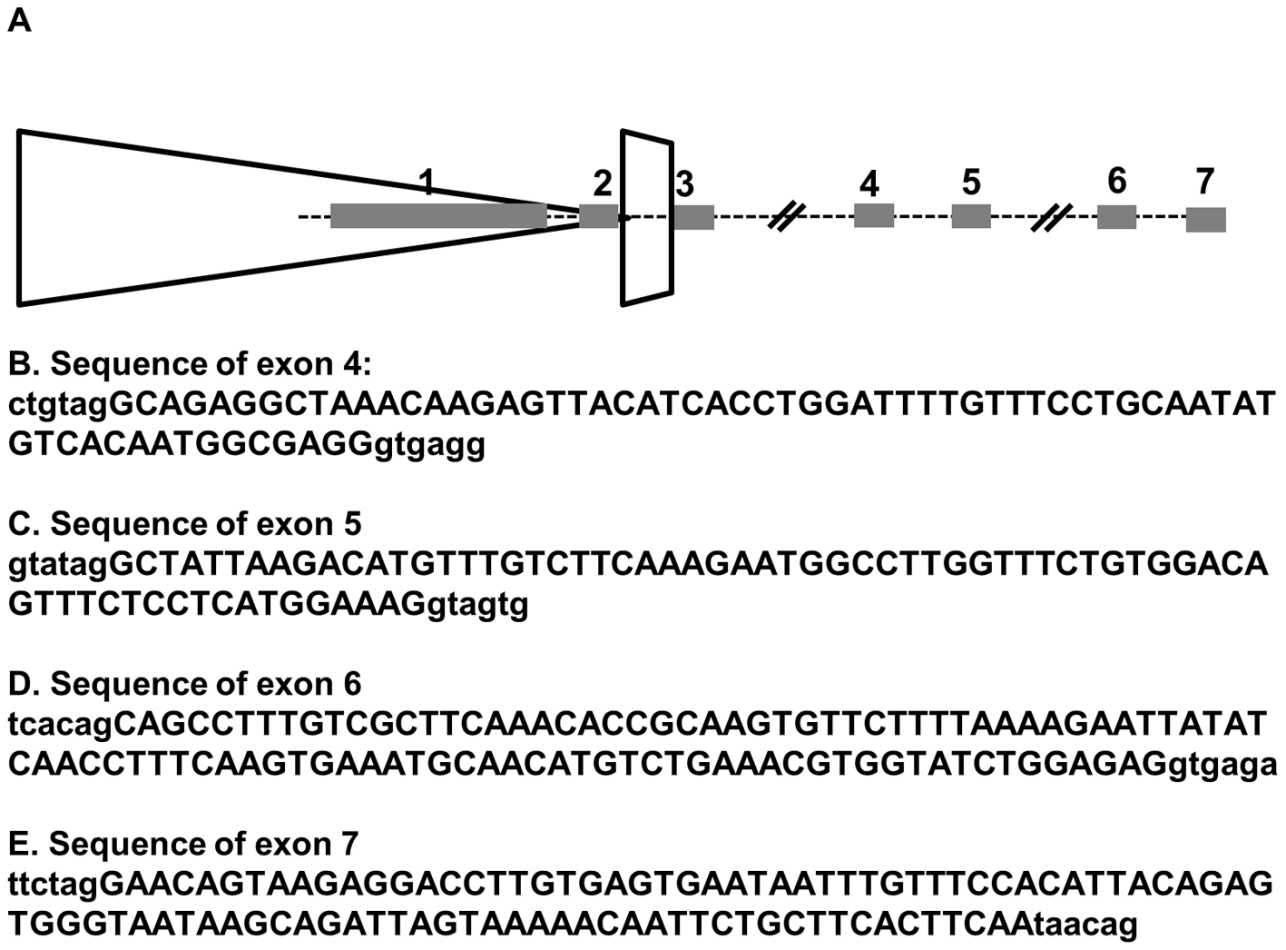
Alternative exon and polyadenylation site usage in germ-line and somatic tissues. (A) Schematic of last D4Z4 unit, last partial repeat, and distal exons. Exon 7 is approximately 6.5 kb from the polyadenylation site in exon 3. DUX4-fl from FSHD muscle contains exons 1-2-3. DUX4-s uses a non-consensus splice donor in the middle of exon 1 to create a short exon 1: 1s-2-3. Both are derived exclusively from chromosome 4A in muscle and other sampled somatic tissues. Germ line tissue expresses the 4A transcript with exons 1-2-3 but also expresses both 4A and 10A transcripts with exons 1-2-6-7. We have also identified a transcript in testis that shows a 1-2-4-5-6-7 splice usage. (B–E) Exon sequences (upper case letters) with flanking genomic sequence (lower case letters). B, Exon 4. C, Exon 5. D. Exon 6. E. Exon 7. Exon 6 and 7 sequences are from a cDNA assigned to chromosome 10, exon 4 and 5 sequences are from a cDNA assigned to 4A161.

3-prime RACE analysis on testis mRNA demonstrated that the chromosome 10 transcripts used alternative 3-prime exons with a polyadenylation signal in exon 7 that is approximately 6.5 kb further telomeric than the previously identified 4A polyadenylation site in the pLAM region (GenBank HQ266763) (Figure 5). Some 4A transcripts also use the exon 7 polyadenylation site (Genbank HQ266764 and HQ266765), but the exon 3 polyadenylation site associated with the permissive allele is preferred (data not shown). The 4B transcripts do not use either the exon 3 or exon 7 polyadenylation sites since the 4B haplotype lacks these regions, however, we have not yet identified the full 3-prime sequence of the DUX4 mRNA from the 4B chromosome. Re-analysis of the muscle cell line, muscle biopsy, and somatic tissue transcripts did not identify any DUX4 mRNA utilizing the exon 7 polyadenylation site from either chromosome 10 or 4, including a control sample with a contraction to 9 copies of D4Z4 on chromosome 10 (biopsy 2318 in Table 1, data not shown). We conclude that chromosome 10 DUX4 transcripts in the testes use a distal exon 7 polyadenylation signal, whereas this region is not used in somatic tissues, even when the chromosome 10 D4Z4 array has contracted to ten repeats. Therefore, polyadenylated DUX4 mRNA from chromosomes 4 and 10 are present in the testis, but only chromosome 4A produces polyadenylated transcripts in somatic tissues.

### Developmental regulation of alternative splicing suppresses DUX4-fl from chromosome 4

**Figure 6:**
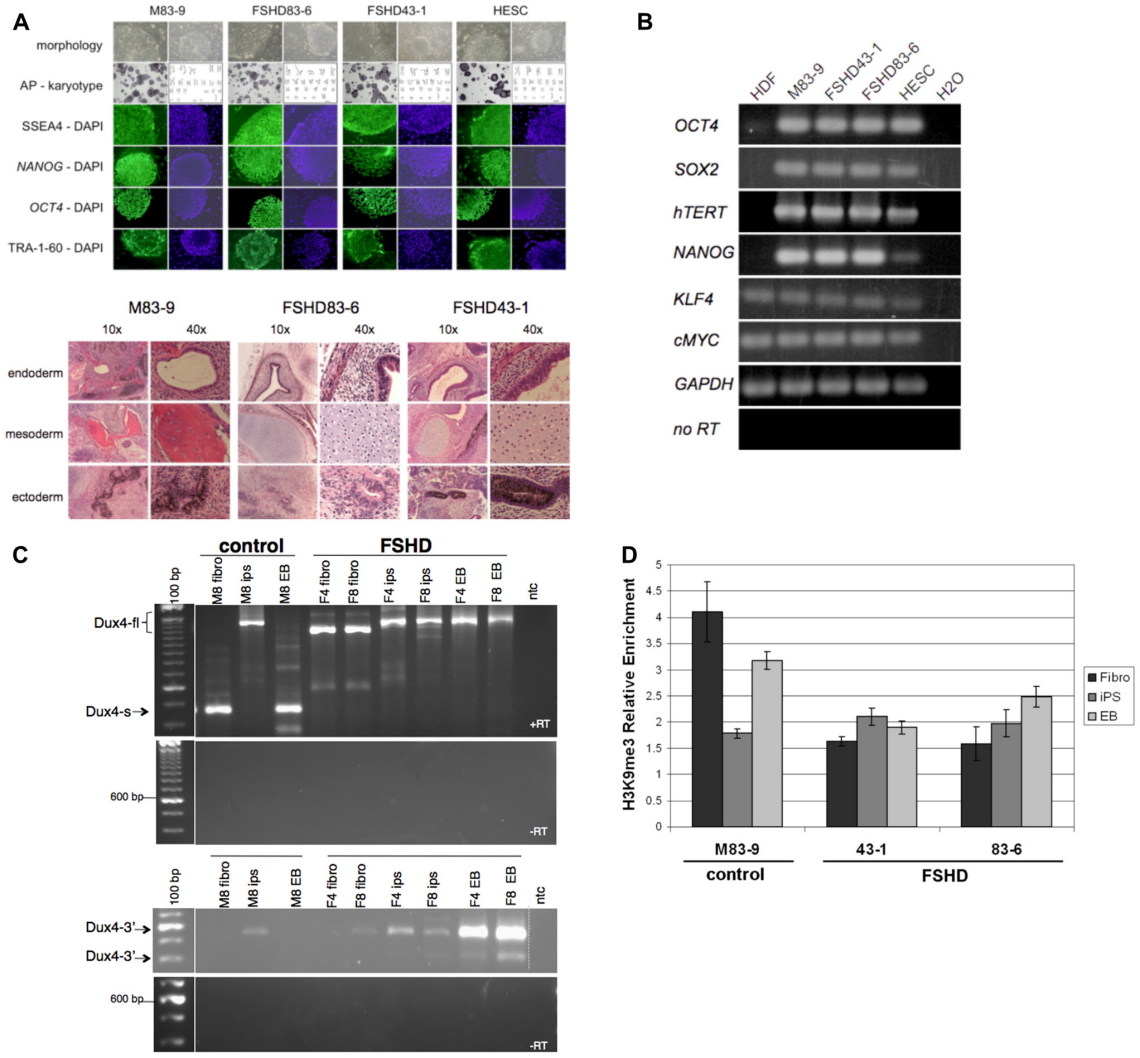
Expression of DUX4-fl and DUX4-s in pluripotent stem cells and differentiated tissues. (A) Top panel shows Induced Pluripotent Stem (iPS) cells from unaffected and FSHD-affected individuals. Human iPS cell lines were generated from skin fibroblasts cultured from an unaffected (M83-9) and two FSHD-affected individuals (FSHD83-6 and FSHD43-1). Human ES cells (HESC) are also shown. Each iPS cell line was generated by transduction of fibroblasts with murine retrovirus vectors encoding Human *SOX2*, *OCT4*, and *KLF4*. Colonies developed approximately 20 days after infection and had the characteristic growth morphology of an iPS cell with flat, well organized colonies, sharply defined colony and cell borders, high nuclear to cytoplasmic ratio, and prominent single nucleoli. Cells contained tissue non-specific alkaline phosphatase activity (AP), had normal karyotypes, and were immunoreactive (green) for Stage Specific Embryonic Antigen 4 (SSEA4), *NANOG*, *OCT4*, and TRA-1-60. 4,6-Diamidino-2-phenylindole (DAPI) staining (blue) indicates total cell content per image. Bottom panel shows hematoxylin and eosin stained tissue sections of teratomas. Teratomas that developed in SCID-Beige mice after intramuscular injection of iPS cells generated from skin fibroblasts of a normal individual (M83-9) or two different FSHD-affected individuals (FHSD83-6 and FSHD43-1). Endoderm-derived tissue is identified by a gut-like structure surrounded by smooth muscle, parenchymal tissue, and lined with a columnar endothelium. Mesoderm-derived tissue is identified by bone (M83-9) or by the presence of cartilage containing chondrocytes (FSHD83-6 and FSHD43-1), and ectoderm-derived tissue is identified by the presence of pigmented neural epithelium (M83-9 and FSHD43-1) or neural rosettes (FSHD83-6). (B) Total cellular RNA was purified from human dermal fibroblasts (HDF), iPS cells used in this study (M83-9, FSHD83-6, FSHD43-1), and Human ES cells (HESC). The presence of RNA transcripts from the genes indicated was detected by priming reverse transcription reactions with oligo dT and PCR amplification of cDNA with oligonucleotides complementary to the sequence of the genes listed (28 cycles). Priming oligonucleotides used for *OCT4*, *SOX2*, and *KLF4* amplification were specific for non-vector encoded transcripts. As a positive control, RNA from Human embryonic stem cells (HESC) was processed in parallel. Water instead of RNA was used as a negative control, and reverse transcriptase was left out of the cDNA synthesis step (-RT) to demonstrate RNA purity, and RNA transcripts from glyceraldehyde phosphate dehydrogenase (*GAPDH*) were amplified to demonstrate RNA integrity. (C) RT-PCR of DUX4 mRNA in iPS cells derived from control or FSHD fibroblasts. M8 (M83-9), control fibroblast line; F4 (FSHD 43-1) and F8 (FSHD 83-6), FSHD fibroblast line. (D) Chromatin immunoprecipitation (ChIP) analysis of H3K9me3 at the 5′-region of DUX4 in control and FSHD fibroblasts, induced pluripotent stem (iPS) cells, and embryoid bodies (EB) differentiated from the iPS. Bars represent relative enrichment by real-time PCR with primers previously described and confirmed as specific to D4Z4. The H3K9me3 IP signals were normalized to control IgG IP and to input, presented as mean ± stdev.

The expression of DUX4-fl mRNA in unaffected human testes and the expression of DUX4-s in some unaffected somatic tissues, including skeletal muscle, suggested a developmental regulation of splice site usage in the DUX4 transcript. To directly determine whether the transition between DUX4-fl and DUX4-s expression is developmentally regulated, we generated induced pluripotent stem (iPS) cells from FSHD and control fibroblasts by expression of *SOX2*, *OCT4*, and *KLF4* transcription factors from Moloney murine leukemia virus vectors [15]. Stem-cell clones had normal karyotypes, exhibited the expected cellular and colony morphology, contained tissue non-specific alkaline phosphatase activity, and expressed embryonic antigens (Figure 6A). RT-PCR demonstrated expression of stem cell markers *NANOG*, *HTERT*, *cMYC*, and endogenous transcripts from *OCT4*, *SOX2*, and *KLF4* (Figure 6B). Pluripotency was demonstrated by the ability to form teratomas containing tissues derived from ectoderm, endoderm, and mesoderm (See Figure 6A). We used these characterized iPS cells to determine the expression of DUX4-fl and DUX4-s in the parental fibroblasts, undifferentiated iPS cells, and in the iPS cells after differentiation into embryoid bodies.

DUX4-s, but not DUX4-fl, was detected in control fibroblasts. In contrast, iPS cells derived from the control fibroblasts expressed DUX4-fl, whereas differentiation of these cells to embryoid bodies resulted in a switch to the expression of DUX4-s and loss of DUX4-fl transcripts (Table 2 and Figure 6C). In contrast, DUX4-fl was detected in FSHD fibroblasts and the iPS cells and embryoid bodies derived from FSHD fibroblasts. As expected, DUX4-fl3′ was detected in samples expressing DUX4-fl. (The relative amounts of DUX4-fl in a subset of iPS cells is shown in Figure 3D and a band migrating at the size of DUX4 was detected on a western with an anti-DUX4 antibody (data not shown)). DUX4-fl was detected in some human ES cell lines, but at much lower levels compared to the iPS cells (data not shown).

All of the splice donor and acceptor sites in the multiple alternative splicing events in the 3-prime UTR have consensus splice donor and acceptor sequences. In contrast, the splice donor in the ORF that produces DUX4-s is a non-canonical donor sequence and would normally not be favored for splicing. Recent studies have indicated that repressive chromatin modifications can favor splice donor usage [16] and we tested whether the degree of H3K9me3 correlated with the usage of the DUX4-s splice site. Chromatin immunoprecipitation showed that the control fibroblasts and embryoid bodies with DUX4-s expression had relatively higher levels of trimethylation of lysine 9 in histone H3 (H3K9me3), a repressive chromatin modification, compared to the control iPS cells, which express DUX4-fl (Figure 6D). The FSHD cells maintained relatively low levels of H3K9me3 in both iPS and differentiated cells. These findings are consistent with previous studies showing decreased H3K9me3 at the D4Z4 region in FSHD1 and FSHD2 [3] and suggest a correlation between the relatively higher levels of repressive chromatin modifications and the use of the cryptic splice donor to produce DUX4-s.

## Discussion

We note that prior studies reported the presence of polyadenylated DUX4 transcripts in a small number of samples of cultured FSHD muscle cells but not in control muscle cells [7], [8]. Our study both confirms and significantly extends these prior studies by (a) including a larger number of FSHD muscle cell cultures, (b) assaying controls that have a permissive 4A chromosome and non-permissive 4B chromosomes, (c) extending the analysis to mRNA from primary muscle biopsies of FSHD and haplotype-matched controls, (d) identifying the DUX4-s splice form of the DUX4 mRNA in control cells and showing that the qualitative difference between control and affected muscle is splice-site usage and not production of DUX4 mRNA; (e) demonstrating that the very low abundance of DUX4 mRNA in FSHD muscle represents a small percentage of nuclei with relatively high abundance mRNA and protein; (f) demonstrating that relatively high amounts of the DUX4 mRNA are expressed in the human testes and pluripotent cells and that developmental regulation is achieved by a combination of chromatin-associated splice-site usage and polyadenylation site usage.

Together our data provide the basis for a specific model of FSHD pathophysiology: (1) full-length DUX4 is produced from the last D4Z4 unit in early stem cells; (2) in differentiated tissues, the D4Z4 array is associated with increased repressive H3K9me3 and DUX4 expression is repressed; (3) in the residual transcripts that escape repression, an alternative first-intron splice donor is utilized to produce DUX4-s instead of DUX4-fl; (4) contraction of the D4Z4 arrays impedes the conversion to repressive chromatin and the transition from DUX4-fl to DUX4-s, resulting in expression of the full-length DUX4 in skeletal muscle and possibly other tissues; and (5) the very low levels of full-length DUX4 expression in FSHD muscle reflects relatively high amounts of expression in a small sub-population of cells. Several groups have shown that expression of full-length DUX4 in muscle cells can induce pathologic features of apoptosis and expression of PITX1 [7], [11], [17], [18]. In contrast, expression of DUX4c, a DUX4-like protein that lacks the carboxyterminal portion of DUX4, does not induce apoptosis [18]. Therefore, it is reasonable to believe that expression of DUX4-fl might induce muscle cell damage in FSHD, whereas DUX4-s expression would not be harmful to the cells. Indeed, FSHD muscle cells expressing the endogenous DUX4 have nuclear foci of DUX4 characteristic of the foci that appear during early stages of apoptosis when DUX4 is exogenously expressed in human skeletal muscle cells (see Figure 2), suggesting, but not yet proving, that these DUX4 expressing cells might be initiating a process of nuclear death.

The observed association of decreased H3K9me3 of D4Z4 with detectable levels of DUX4-fl mRNA suggests a specific mechanism of regulating DUX4 splicing. Previously [9], we demonstrated bidirectional transcription of the D4Z4 repeats with the generation of small si/mi/pi-like RNA fragments and suggested that the small RNAs generated from D4Z4 might function to suppress DUX4 expression in a developmental context, a suppression mechanism observed for other retrogenes [19], [20], [21]. A recent publication demonstrated that the small RNAs mediating heterochromatin formation also regulate splice-donor usage, either by targeting the nascent transcripts or by altering the rate of polymerase progression through condensed chromatin [16], [22]. Therefore, the repressive chromatin associated with D4Z4 in differentiated cells might facilitate the usage of the non-canonical splice donor to generate DUX4s, either through siRNAs from the region or through the impediment of polymerase progression, whereas the more permissive chromatin in FSHD and pluripotent cells might favor polymerase progression through to the consensus splice donor and generate DUX4-fl.

A recent study by Lemmers et al [8] identifies sequence variants on 4A necessary to produce polyadenylated DUX4 mRNA transcripts in somatic tissues. Our results are consistent with these findings since we have not been able to identify polyadenylated transcripts from non-permissive alleles in somatic tissues. In contrast, we do find alternative distal polyadenylation usage for DUX4 mRNA from non-permissive alleles in the testis. Developmentally regulated polyadenylation site usage has been described for other genes [23] and appears to be one additional mechanism of silencing expression of the DUX4 retrogene in somatic cells.

Our finding that the wild-type chromosomes 4 and 10 express a full-length DUX4 mRNA in human testes, most likely in the germ-line, and that the protein is relatively abundant suggests that DUX4 might have a normal role in development. This is supported by the expression of canine DUXC in germ-line tissue (L. Geng, unpublished data). In addition, a DUX4-like gene in the mouse, Duxbl, is expressed in mouse germ-line cells in both spermatogenesis and oogenesis, as well as in early phases of skeletal muscle development [13]. Similar to DUX4, Duxbl has developmentally regulated splicing to produce a full-length protein and a protein truncated after the double homoeodomains and studying the roles of Duxbl in germ-line and muscle development in mouse will likely inform our understanding of DUX4. We should note that our study describes the expression of human DUX4 in testes but we believe it is likely to be expressed in oogenesis as well. Limited access to appropriate tissue has limited our ability to carefully examine expression in cells of the ovary.

Generating new genes through retrotransposition is a common mechanism of mammalian evolution [24], particularly for genes with a role in germ cell development. Recently an FGF4 retrogene was identified as causing the short-legged phenotype in many dog breeds [25], indicating that retrogenes can direct dramatic phenotypic evolution in a population. Our study demonstrates that the expression of the DUX4 retrogene is developmentally regulated and might have a role in germ-line development, and, if similar to Duxbl, possibly in aspects of early embryonic muscle development. Maintaining the DUX4 retrogene in the primate lineage suggests some selective advantage compared to maintaining the parental gene itself. Based on current knowledge, this could be due to a function in germ-line development, or to a modulation of muscle mass in primate face and upper extremity. In this regard, it is interesting to speculate that a normal function of the DUX4 retrogene might be to a regulate the development of facial and upper-extremity muscle mass in the primates, and that FSHD represents a hypermorphic phenotype secondary to inefficient developmental suppression. Alternatively, the persistent expression of full-length DUX4 might induce a neomorphic phenotype unrelated to an evolutionarily selected role of DUX4. In either case, our findings substantiate a comprehensive developmental model of FSHD and demonstrate that FSHD represents the first human disease to be associated with the incomplete developmental silencing of a retrogene array that is expressed in pluripotent stem cells and in normal development.

## Materials and Methods

### Ethics statement

This study used pre-existing and de-identified human tissue samples from tissue repositories and commercial sources and was approved by the Fred Hutchinson Cancer Research Center and the University of Washingtion Institutional Review Boards. Animal studies were approved by the University of Washington Institutional Animal Care and Use Committee and followed the Assessment and Accreditation of Laboratory Animal Care guidelines.

### Muscle biopsies, cultures, and human RNA and protein

Muscle biopsy samples were collected from the vastus lateralis muscle of clinically affected and control individuals using standardized needle muscle biopsy protocol and cell cultures were derived from biopsies as described on the Fields Center website: http://www.urmc.rochester.edu/fields-center/protocols/documents/PreparingPrimaryMyoblastCultures.pdf. The sex, age, and severity score for the FSHD muscle biopsies were: F1998 (M, 43, 2); F0519 (M, 43, 4); F0515 (F, 48, 2); F0509 (M, 47, 2); F0531 (F, 47, 2); F2306 (F, 46, ND); F2331 (F, 56, 4); F2316 (F, 34, 5); F2319 (M, 52, ND); F2315 (F, 40, 3). Pathologic grading scale is 0–12 (from normal to severe) based on a score of 0–3 for each of four parameters: muscle fiber size/shape; degree of central nucleation; presence of necrotic/regenerating fibers or inflammation; and degree of fibrosis. Controls were selected in the same age range and sex representation. Muscle cell culture MB216 and muscle biopsy F2316 are from the same individual, otherwise the muscle cultures were derived from other individuals. RNA and protein lysates from human tissues were purchased from BioChain (Hayward, CA) and Origene (Rockville, MD).

### RT-PCR for DUX4-fl, DUX4-s, and DUX4-fl3′

Total RNA was isolated from muscle biopsies and cultured cells using Trizol (Invitrogen) and then treated with DNase I for 15 minutes using conditions recommended by Invitrogen with the addition of RNaseOUT (Invitrogen) to the reaction. DNase reaction components were removed using the RNeasy (Qiagen) system and RNA eluted by two sequential applications of 30 µl of RNase-free water. Volume was reduced by speed vac and 1.5–2 µg of RNA used for first strand cDNA synthesis. RNA from adult human tissues was purchased from Biochain and had been DNase-treated by the supplier. First strand synthesis was performed using Invitrogen SuperScript III reverse transcriptase and Oligo dT primers according to manufacturer's instructions at 55° for 1 hour followed by digestion with RNase H for 20 minutes at 37°. Finally, the reactions were cleaned using the Qiaquick (Qiagen) pcr purification system and eluted with 50 µl of water. Primary pcr reactions were performed with 10% Invitrogen PCRx enhancer solution and Platinum Taq polymerase using 10–20% of the first strand reaction as template in a total reaction volume of 20 µl in thin wall MicroAmp (Applied Biosystems) reaction tubes. Nested pcr reactions used 1 µl of the primary reaction as template. Primers for Dux4-fl and -s detection in biopsy and cultured cell samples were 14A forward and 174 reverse, nested with15A (or 16A) forward and 175 reverse. Primers for 3′ detection were 182 forward and 183 reverse nested with1A forward and 184 reverse. All primer sequences are listed in Table 5.

**Table 5:** Primer sequences.

1A	5′ GAGCTCCTGGCGAGCCCGGAGTTTCTG 3′	forward
14A	5′ CCCCGAGCCAAAGCGAGGCCCTGCGAGCCT 3′	forward
15A	5′ CGGCCCTGGCCCGGGAGACGCGGCCCGC 3′	forward
16A	5′ GGATTCAGATCTGGTTTCAGAATCGAAGG 3′	forward
92	5′ CAAGGGGTGCTTGCGCCACCCACGT 3′	forward
116	5′ GGGGTGCGCACTGCGCGCAGGT 3′	reverse
133	5′ ATGGCCCTCCCGACACCCTCGGACAGCACC 3′	forward
134	5′ CTCGGACAGCACCCTCCCCGCGGAAGCCCG 3′	forward
135	5′ GGAAGCCCGGGGACGAGGACGGCGACGGAG 3′	forward
136	5′ CTAAAGCTCCTCCAGCAGAGCCCGGTATTCTTCCTC 3′	reverse
137	5′ CCCGGTATTCTTCCTCGCTGAGGGGTGCTTCCAG 3′	reverse
138G	5′ GGGGTGCTTCCAGCGAGGCGGCCTCTTCCG 3′	reverse
138S	5′ CGGAGTTTCTGCAGCAGGCGCAACCTCTCCT 3′	forward
174	5′ GTAACTCTAATCCAGGTTTGCCTAGA 3′	reverse
175	5′ TCTAATCCAGGTTTGCCTAGACAGC 3′	reverse
182	5′ CACTCCCCTGCGGCCTGCTGCTGGATGA 3′	forward
183	5′ CCAGGAGATGTAACTCTAATCCAGGTTTGC 3′	reverse
184	5′ GTAACTCTAATCCAGGTTTGCCTAGACAGC 3′	reverse
187	5′ CTGCTGGTACCTGGGCCGGCTCTGGGATCCC 3′	reverse
188	5′ GTACCTGGGCCGGCTCTGGGATCCCCGGGAT 3′	reverse
OCT4OCT4	5′-GACAGGGGCAGGGGAGGAGCTAGG-3′ 5′-CTTCCCTCCAACCAGTTGCCCCAAAC-3′	forwardreverse
SOX2SOX2	5′-GCTAGTCTCCAAGCGACGAA-3′ 5′-GCAAGAAGCCTCTCCTTGAA-3′	forwardreverse
hTERThTERT	5′-CCTGCTCAAGCTGACTCGACACCGTG-3′ 5′-GGAAAAGCTGGCCCTGGGGTGGAGC-3′	forwardreverse
NANOGNANOG	5′-CAGTCTGGACACTGGCTGAA-3′ 5′-CTCGCTGATTAGGCTCCAAC-3′	forwardreverse
KLF4KLF4	5′-TATGACCCACACTGCCAGAA-3′ 5′-TGGGAACTTGACCATGATTG-3′	forwardreverse
cMYCcMYC	5′-CGGAACTCTTGTGCGTAAGG-3′ 5′-CTCAGCCAAGGTTGTGAGGT-3′	forwardreverse
GAPDHGAPDH	5′-TGTTGCCATCAATGACCCCTT-3′ 5′-CTCCACGACGTACTCAGCG-3′	forwardreverse

Dux4-fl and -s in adult human tissues were detected using 14A forward and 183 reverse, then nested with 15A forward and 184 reverse primers. Pcr cycling conditions were as follows for both primary and nested pcr: 94° 5 minutes denaturation, 35 cycles of 94° for 30″, 62° for 30″ and 68° for 2.5 minutes or 1 minute depending on expected length of product. A single final extension of 7 minutes at 68° was included. Pcr products were examined on 2% NuSieve GTG (Lonza) agarose gels in TBE.

### Pooled PCR for DUX4

To assess for stochastic expression of DUX4 in affected muscle cells, FSHD primary myoblasts were trypsinized and collected at confluence or after differentiation for 96 hr. Cells were counted and split into pools of 100-cell, 600-cell, or 10,000-cell aliquots. RNA was extracted from individual aliquots using Dynabeads mRNA DIRECT Kit (Invitrogen) following manufacturer's instructions. Bound polyadenylated mRNA was used directly for reverse transcription reaction with SuperScript III using on-bead oligo dT as primer. Synthesis was carried out at 52°C for 1 hr, terminated at 70°C for 15 min, followed by 15 min of RNase H treatment. 2 uL of cDNA product was used for nested DUX4-fl3′ PCR as described above.

### RT-PCR for transcripts from chromosomes 10 and 4

Pcr reactions were performed on RT reactions generated as described above and using nested primer sets to sequences in exons 1 and 2 that are common to alleles on chromosomes 4 as well as 10. Transcripts were detected using primers 1A and 187 followed by nesting with 138S and 188 (Table 5). Diagnostic polymorphisms (underlined) in the 5′ end of exon 2 were used to assign allele origins of transcripts:

### Quantitative RT-PCR for DUX4-fl3′

For quantitative PCR, 1 ug of DNase'd RNA was used for first strand cDNA synthesis. Reverse transcription was performed as above, except at 52°C for the synthesis reaction followed by 15 minutes of RNase H treatment and the Qiaquick purification eluted in 30 µl of water. One round of PCR reactions were performed using the same reagents as above and 2 uL of purified cDNA template. Primers for full length detection were 92 forward and 116 reverse (Table 5). PCR cycling conditions were as follows: 95°C 5 min denaturation, 36 cycles of 95°C for 30″, 62°C for 30″ and 68°C for 1 min, and final extension of 5 min at 68°C. Sequence of the product matched DUX4. A standard curve for DUX4 template copies was generated from PCR reactions using the same primers and cycling conditions but with known dilutions of a plasmid containing full length DUX4 cDNA in water. Test sample PCR reactions and standard PCR reactions were run in triplicate and examined on the same 1% agarose/TBE gels stained with SYBR Gold (Invitrogen) for 40 min per manufacturer instructions. Fluorescence was detected with Typhoon Trio Multi-mode Imager (GE Healthcare): excitation laser 488 nm; emission filter 520DP 40, PMT 500 V, 100 µm resolution. Histogram analysis was performed to ensure no signals were saturated. Gel band intensities were quantified with ImageQuant TL v2005 (GE Healthcare) software. Estimates for the copies of DUX4 full length template in the test samples were interpolated from the line of best fit of the dilutional standards, with the lowest visible dilutional signal setting the detection limit. The interpolated number was doubled to adjust for the single-stranded cDNA input in contrast to the double-stranded plasmid standard input. This resulted in an estimated copy number of DUX4 full-length per ug of total RNA. Final copy number estimates per cell were calculated based on assumptions of 100% efficient reverse transcription and 3.3 pg of total RNA per cell.

### Open reading frame PCR for DUX4-fl

To assess for the full coding region of DUX4, three rounds of PCR were performed on cDNA, totaling 36 cycles. Conditions for each round were as follows: 95°C for 5′, 3 cycles of 95°C for 30″ and 68°C for 1′33″, 3 cycles of 95°C for 30″ and 65°C for 30″ and 68°C for 1′33″, 6 cycles of 95°C for 30″ and 62°C for 30″ and 68°C for 1′33″. 3 uL of primary PCR was used in the secondary PCR, and 3 uL of secondary PCR were used in the tertiary PCR. Primers for successive rounds of pcr (133, 134, 135, 136, 137, and 138G) are listed in Table 5.

### 3′ RACE for DUX4 in human testes

3′ RACE was performed on total RNA using Invitrogen Gene Racer kit essentially as described. Prior to pcr with gene specific primers and the GeneRacer 3′ primers the RT reaction was cleaned using Qiaquick (Qiagen) spin columns as described above. Gene specific forward primers were 182 and 1A (nesting). Pcr products were gel purified, cloned into TOPO 4.0 (Invitrogen) and sequenced.

### Generation of induced pluripotent stem (iPS) cells

iPS cells were generated by forced expression of human OCT4, SOX2, and KLF4 using the retroviral vectors essentially as previously described (1). MLV vectors (pMXs-hOCT4, pMXs-hSOX2, and pMXs-hKLF4) were purchased from Addgene (www.addgene.com, Cambridge, MA) and vector preparations were generated by transient transfection of Phoenix-GP cells (2) with pCI-VSV-G and vector plasmids (1∶1 ratio), replacing the culture medium 16 and 48 hours later, harvesting and filtering (0.45 um pore size) conditioned medium after a 16 hour exposure to cells, and concentrating 50 to 100-fold by centrifugation (3). Transduction with MLV vectors was performed with polybrene (4ug/ml concentration) (Sigma-Aldrich Corp., St. Louis, MO) added to the medium. iPS cell colonies were identified by their characteristic morphology, cloned by microdissection, and expanded on irradiated mouse embryo fibroblasts (6000 rads) for further characterization. Typically, 5×10^4^ fibroblasts cultured in DMEM plus 10% FBS were seeded to a 9.4 cm^2^ well on day minus 1, the medium was replaced with medium containing vectors and polybrene on day 0, and changed again to medium with DMEM plus 10% FBS on day 1. Cells were detached with trypsin and seeded to five 55 cm^2^ dishes on day 2 and medium changed on day 4. On day 6 cells are again detached with trypsin and 5×10^5^ cells seeded to 55 cm^2^ dishes containing 7×10^5^ irradiated mouse embryo fibroblasts (6000 rads) in human ES cell culture medium (see below). Medium is replaced every other day and colonies with typical morphology of iPS cells appear between day 20 and day 30 post infection. Colonies are mechanically dissected using drawn Pasteur pipettes and seeded to mouse embryo fibroblast feeder layers for culture and passaged every 2–3 days using 2 u/ml dispase.

### Stem cell culture

iPS cells and Human ES cells were grown in a solution of DMEM∶F12 (1∶1) with 3.151 g/L glucose, supplemented with L-Glutamine (Invitrogen), non-essential amino acids (10 mM (100×) liquid, Invitrogen, # 11140-076), sodium pyruvate (100 mM (100×), liquid, # 11360-070), 20% knockout serum replacer (# 10828010) (Invitrogen, Carlsbad, CA), 1mM beta-mercapto-ethanol (Sigma, St. Louis MO), and 5 ng/ml basic fibroblast growth factor (Peprotech, #AF-100-18B ). Cells were generally cultured in 0.1% gelatin coated dishes containing irradiated mouse embryo fibroblasts at a density of 1.3×10^4^ cells/cm^2^. When cells were used as a source of RNA, DNA, or protein, they were cultured on matrigel (1∶60 dilution, BD biosciences, #356234) coated dishes in medium conditioned by exposure to confluent layers of mouse embryo fibroblasts over a 3 day period. Cells were passaged a minimum of 4 times under these conditions before DNA, RNA, or protein was harvested.

### Detection of embryonic antigens in iPS cells

iPS cells were evaluated for the presence of tissue non-specific alkaline phosphatase activity by fixing colonies in phosphate buffered saline solution containing 0.5% gluteraldehyde, and washing ×3 in PBS. A staining buffer containing 100 mM Tris pH 8.5, 100 mM NaCL, 50 mM MgCl_2_, 0.1 mg/ml 5-Bromo-4-chloro-3-indolyl phosphate (xphos) and 1 mg/ml p-Nitro-Blue tetrazolium chloride (NBT) (Sigma-Aldrich, St. Louis, MO, USA) was used to detect tissue non-specific alkaline phosphatase activity. Stage Specific embryonic antigen 4 (SSEA4) was detected using mouse monoclonal MC-813-70 and goat anti-mouse FITC conjugated secondary. TRA-1-60 was detected using mouse monoclonal TRA-1-60 (Millipore, Billerica, MA), and goat anti-mouse FITC conjugated secondary (Millipore, Billerica, MA). Human NANOG was detected with a goat polyclonal flurophore (Northern Lights) conjugated antibody (NL493, R & D systems, Minneapolis, MN). Human OCT4 was detected with a rabbit polyclonal (Abcam, Cambridge, MA) and goat anti rabbit secondary conjugated with the Alexa 488 flurophore (Invitrogen, Carlsbad, CA). Cell karyotypes were determined by the University of Washington Cytogenetics laboratory.

### Teratoma formation and staining

Induced pluripotent stem cells were detached from culture dishes with dispase (2 units/ml working concentration), 2×10^6^ cells resuspended in F12∶DMEM (1∶1 mixture) medium without supplements, and injected into the femoral muscle of SCID-Beige mice (CB17.B6-*Prkd*c^scid^ *Lyst^bg^*/Crl Charles River, Stock # 250). Mice were maintained under biosafety containment level 2 conditions and palpable tumor masses developed approximately 6 weeks later. When a tumor mass was palpable the mice were sacrificed and tumor tissue fixed for several days in phosphate buffered saline solution containing 4% formaldehyde, and imbedded in paraffin. Sections of the tumor (5 micron thickness) were placed on slides and stained with hematoxylin and eosin using standard protocols.

### Embryoid body formation

Human iPS were prepared for embryoid body formation by expanding cell numbers on mouse irradiated feeder layers, detaching colonies with dispase, triturating with a Pasteur pipette, and seeding colony fragments to dense layers of mouse embryo fibroblast feeders (5×10^4^ irradiated mef/cm^2^) prior to EB formation. Four days later densely grown colonies from a 55 cm^2^ dish were treated with dispase and gently detached by pipetting or scraping. Colony fragments were washed several times and seeded (1∶1) to Ultra Low Attachment 55 cm^2^ culture dishes (Corning, Corning, NY) in DMEM supplemented with 20% Fetal Bovine Serum. Every three days, EB's were allowed to gravity settle and the medium was gently removed and replaced. RNA and chromatin was harvested three weeks later for analysis.

### Analysis of gene expression in iPS cells

iPS cells were grown without MEF feeders for preparation of RNA to be used in gene expression analysis. Cells were seeded to matrigel coated dishes and filtered conditioned medium from mouse embryo fibroblasts was used for culture. RNA was purified from cells using standard techniques and treated with DNAse to remove residual genomic DNA from the cells. cDNA synthesis was primed with oligo dT and reverse transcriptase. In all cases a tube was processed in parallel without the addition of reverse transcriptase to serve as a control for possible DNA contamination. The presence of RNA transcripts were detected using 28 thermal cycles with the primer pairs for OCT4, SOX2, hTERT, NANOG, KLF4, cMYC, and GAPDH indicated in Table 5. RNA was replaced with water as a negative control for the reaction.

### Chromatin Immunoprecipitation

The Chromatin Immunoprecipitation (ChIP) analysis of repressive histone modifications at the 5′-region of *DUX4* was performed on primary fibroblasts, induced pluripotent stem (iPS) cells and corresponding embryoid bodies (EB) derived from unaffected individuals and FSHD patients, following a previously described protocol [3], [26]. Briefly, cells were cross-linked with formaldehyde at 1.42% final concentration for 15 min at room temperature, quenched, and sonicated to generate 500–100 bp DNA fragments. 25 µg aliquots (representing approximately 500,000 cells) of chromatin were used for each immunoprecipitation with anti-Histone H3K9me3 antibodies (Abcam) and nonimmune IgG fraction used as a mock control. After reverse cross-linking and DNA purification, the IP products were analyzed by real time PCR. The 5′-region of the DUX4 gene was analyzed using the 4q-specific D4Z4 primers, 4qHox or Q-PCR, that detect internal D4Z4 units including the last repeat unit [3]. The real-time PCR signals obtained for IP antibodies were normalized to mock control IgG and to input to account for the number of D4Z4 repeats. Data are presented as mean ± stdev and represent the results of at least three independent immunoprecipitations followed by real-time PCR analysis done in triplicates.

### Generation of antibodies to DUX4

We generated monoclonal antibodies to the amino- and carboxy-terminus of DUX4 for this study. The full characterization of these antibodies will be published separately [10]. Briefly, the N-terminal 159 amino acids and the C-terminal 76 amino acids of DUX4 were fused to glutathione-s-transferase tags, respectively, and injected into the animals as immunogens. Mouse monoclonals were produced at the Antibody Development core facility at the Fred Hutchinson Cancer Research Center and will be commercially available. Rabbit monoclonals were produced in collaboration with and will be available through Epitomics (Burlingame, CA). Hybridoma clones were screened for specificity by ELISA, western blot and immunofluorescence in C2C12 myoblasts transfected with DUX4. The C-terminal antibodies P4H2, P2B1 and E5-5 are specific to DUX4 and do not recognize DUX4c, whereas the N-terminal antibodies P2G4 and E14-3 recognize both DUX4 and DUX4c.

### Protein analysis

For western blotting, protein lysates were prepared by resuspension in standard Laemmli buffer and sonicated briefly. Equivalent amounts of test samples were loaded onto 4–12% gradient gel and transferred to nitrocellulose membrane, which were then blocked with 5% non-fat dry milk in PBS 0.1% Tween-20. Custom monoclonal antibodies (Epitomics, Burlington, CA) raised against DUX4 were used to probe the blots and detected by ECL reagent (Pierce, Rockford, IL). Membranes were stripped and reprobed with anti-α-tubulin antibody (Sigma-Aldrich, St Louis, MO) for loading control. Immunoprecipitation was performed on samples resuspended in PBS with protease inhibitor cocktail (Roche) by incubating overnight at 4°C with pooled anti-DUX4 rabbit monoclonal antibodies bound to protein A- and G-coupled Dynabeads (Invitrogen, Carlsbad, CA). Samples were eluted directly into Laemmli buffer and analyzed on western blot as described. For immunofluorescence, cells were fixed in 2% paraformaldehyde for 7 min and permeabilized in 1% Triton X-100 in PBS for 10 min at room temperature. Cells were probed with pairs of rabbit and mouse primary antibodies raised against N- or C-terminus of DUX4 diluted in PBS overnight at 4°C. Double labeling was detected with Alexa Fluor 488 goat anti-mouse IgG and Alexa Fluor 568 goat anti-rabbit IgG (Invitrogen) at 1∶500 in PBS for 1 hr and counterstained with DAPI.

### DUX4 IHC on frozen tissue

Immunohistochemistry was performed by the FHCRC Experimental Histopathology Shared Resource. Six-micron sections of OCT embedded frozen de-identified human testes tissue were sectioned and fixed for 10 minutes in 10% neutral buffer formalin. The slides were rehydrated in TBS-T wash buffer, permeablized with 0.1% triton X-100 for 10 minutes, and then endogenous peroxidase activity was blocked with 0.3% hydrogen peroxide (Dako, Carpinteria, CA) for 8 minutes. Five minute incubation in 50% acetone and 50% methanol was used for antigen retrieval on a subset of slides. Protein block containing 0.25% casein and 0.1% Tween 20 was applied for 10 minutes. Slides were incubated over night at 4 degrees C with a 1∶5 dilution of either clone E5-5 or P2B1 in a 0.3 M NaCl antibody diluent containing 1% BSA. Staining was developed using Mach2 HRP-labeled polymers (Biocare Medical, Concord, CA). The staining was visualized with 3,3′-diaminobenzidine (DAB, Dako, Cupertino, CA) for 8 minutes, and the sections were counter-stained with hematoxylin (Dako) for 2 minutes. Concentration matched isotype control slides were run for each tissue sample (Jackson ImmunoResearch).
